# Effects of Eutrophication, Seasonality and Macrofouling on the Diversity of Bacterial Biofilms in Equatorial Coral Reefs

**DOI:** 10.1371/journal.pone.0039951

**Published:** 2012-07-06

**Authors:** Yvonne Sawall, Claudio Richter, Alban Ramette

**Affiliations:** 1 Leibniz Center for Tropical Marine Ecology, Bremen, Germany; 2 Alfred Wegener Institute for Polar and Marine Research, Bremerhaven, Germany; 3 HGF-MPG Group for Deep Sea Ecology and Technology, Max Planck Institute for Marine Microbiology, Bremen, Germany; Argonne National Laboratory, United States of America

## Abstract

Biofilms play an important role as a settlement cue for invertebrate larvae and significantly contribute to the nutrient turnover in aquatic ecosystems. Nevertheless, little is known about how biofilm community structure generally responds to environmental changes. This study aimed to identify patterns of bacterial dynamics in coral reef biofilms in response to associated macrofouling community structure, microhabitat (exposed vs. sheltered), seasonality, and eutrophication. Settlement tiles were deployed at four reefs along a cross-shelf eutrophication gradient and were exchanged every 4 months over 20 months. The fouling community composition on the tiles was recorded and the bacterial community structure was assessed with the community fingerprinting technique Automated Ribosomal Intergenic Spacer Analysis (ARISA). Bacterial operational taxonomic unit (OTU) number was higher on exposed tiles, where the fouling community was homogenous and algae-dominated, than in sheltered habitats, which were occupied by a variety of filter feeders. Furthermore, OTU number was also highest in eutrophied near-shore reefs, while seasonal variations in community structure were most pronounced in the oligotrophic mid-shelf reef. In contrast, the macrofouling community structure did not change significantly with seasons. Changes in bacterial community patterns were mostly affected by microhabitat, seasonal and anthropogenically derived changes in nutrient availability, and to a lesser extent by changes in the macrofouling community structure. Path analysis revealed a complex interplay of various environmental and biological factors explaining the spatial and temporal variations in bacterial biofilm communities under natural conditions.

## Introduction

Microbial biofilms play an important role in aquatic systems by providing a conditioned surface for larval settlement and metamorphosis of sessile organisms [1] and by contributing to nutrient turnover and productivity [2]–[3]. Biofilms generally have a high microbial diversity, which is maintained by exogenous [4] as well as endogenous [3] mechanisms. Exogenous drivers may consist of either a top-down control by predation or viral lysis of bacteria, which limits the dominance of certain species in the community and would allow for the co-existence of different species within the same niche, or a bottom-up control may consist of the wide variety of energy sources and substrates available in an ecosystem, which offer a large variety of niches for bacteria [4]. Endogenous mechanisms include interactions among microbial species, with dynamic exchanges of metabolites, which thereby further contribute to the formation of various ecological niches [3].

Although diversity is generally high, biofilm community structure can greatly vary with changes in environmental conditions [5]–[6], such as nutrient availability, temperature, salinity and light, which can moreover fluctuate over space and time [2], [7]–[9]. Nutrient availability was found to be one of the major factors affecting biofilm diversity and composition (reviewed by [2]), and to vary with seasons [7], [10] or with human impact, i.e. eutrophication [9], [11]–[12]. Higher nutrients generally cause a shift from autotrophic to heterotrophic and to sulphur reducing bacteria as a response to decreased light availability and increased load in organic material [9], [11], [13], while the overall biofilm diversity has been found to either remain unaffected [8], [14] or to increase [12], [15].

Those changes in the biofilm community may further affect the behaviour and success of larval settlement of sessile macroorganisms [16]–[19]. Conversely, macroorganisms reacting to environmental changes may modify their chemical composition and consequently affect their associated bacterial community [20]–[21]. Negri et al. [22] demonstrated that the algae encrusted substrate affected the overlying bacteria, which further influenced the settlement of coral larvae in laboratory experiments [22]. These studies demonstrate well the interactions between biofilm composition and macrofouling, where initial biofilm formation influences the settlement of macroorganisms and the macroorganisms may in turn affect biofilm formation (see also [23]–[24]). However, these studies were all species-specific and so far did not consider community level approaches so as to assess the interplay between microbial and macrobial communities (assembly of fleshy and calcareous algae, bryozoans, ascidicans, barnacles, spirorbid worms, etc.). Furthermore, biofilm studies on hard substratum in coral reefs are scarce. To the best of our knowledge, our study is the first attempt to date to simultaneously address the interactions of microbial biofilms and macrofouling communities in coral reefs in response to spatio-temporal changes in the environment. Understanding these dynamics is of increasing importance in the context of anthropogenic water quality changes, which may strongly affect the interplay of the two communities and consequently the stability of the coral reef ecosystem.

**Figure 1:**
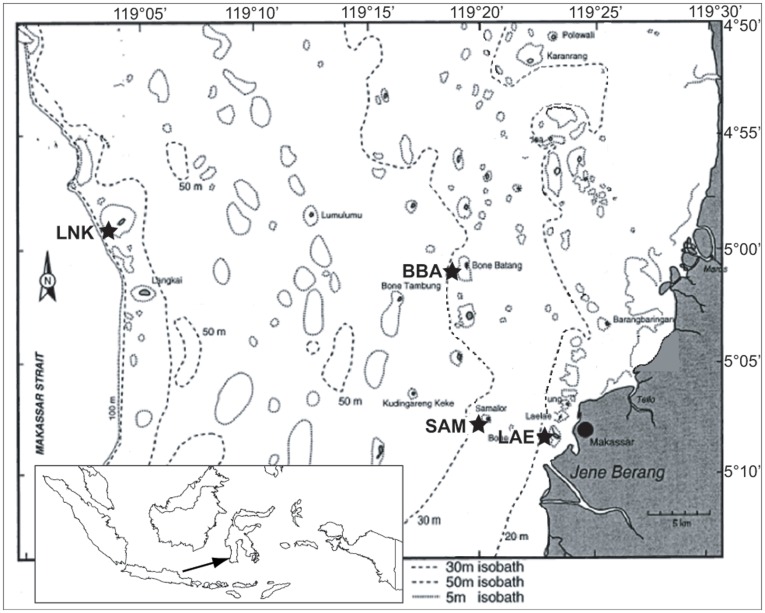
Map of the Spermonde Archipelago, SW Sulawesi (Indonesia) with the study sites indicated by stars: Near-shore Lae Lae (LAE), near mid-shelf Samalona (SAM), mid-shelf Bonebatang (BBA) and off-shore Lanyukan (LNK).

Here, the diversity and dynamics of colonizing bacterial communities were investigated on tiles, which were deployed so as to create sheltered and exposed microhabitats in several coral reefs of the Spermonde Archipelago, Indonesia, over 20 months (Fig. 1). Spermonde is characterized by an eutrophication gradient between nutrient-rich coastal and oligotrophic offshore waters and by seasonal changes in nutrient input and turbidity mainly due to variations in rain fall. The macrofouling community that settled on the tiles was included in the analyses to examine its relationship with biofilm diversity and dynamics. The following main questions were addressed: (1) How much do eutrophication, geographic location and seasonality affect bacterial diversity and community structure? (2) How important is the presence and composition of the macrofouling community for microbial dynamics in the context of microhabitats, eutrophication and seasonality? (3) What effects do microhabitats have on the bacterial community structure? By disentangling the respective effects of space, season, and environmental parameters by using a combination of multivariate statistical tools, a community ecology approach was applied to examine the principles underlying the assembly and shifts of the microbial community and its reciprocal interactions with the macrofouling communities in their natural environment.

## Results

### Spatial Variation in Benthic Community Structure

**Figure 2:**
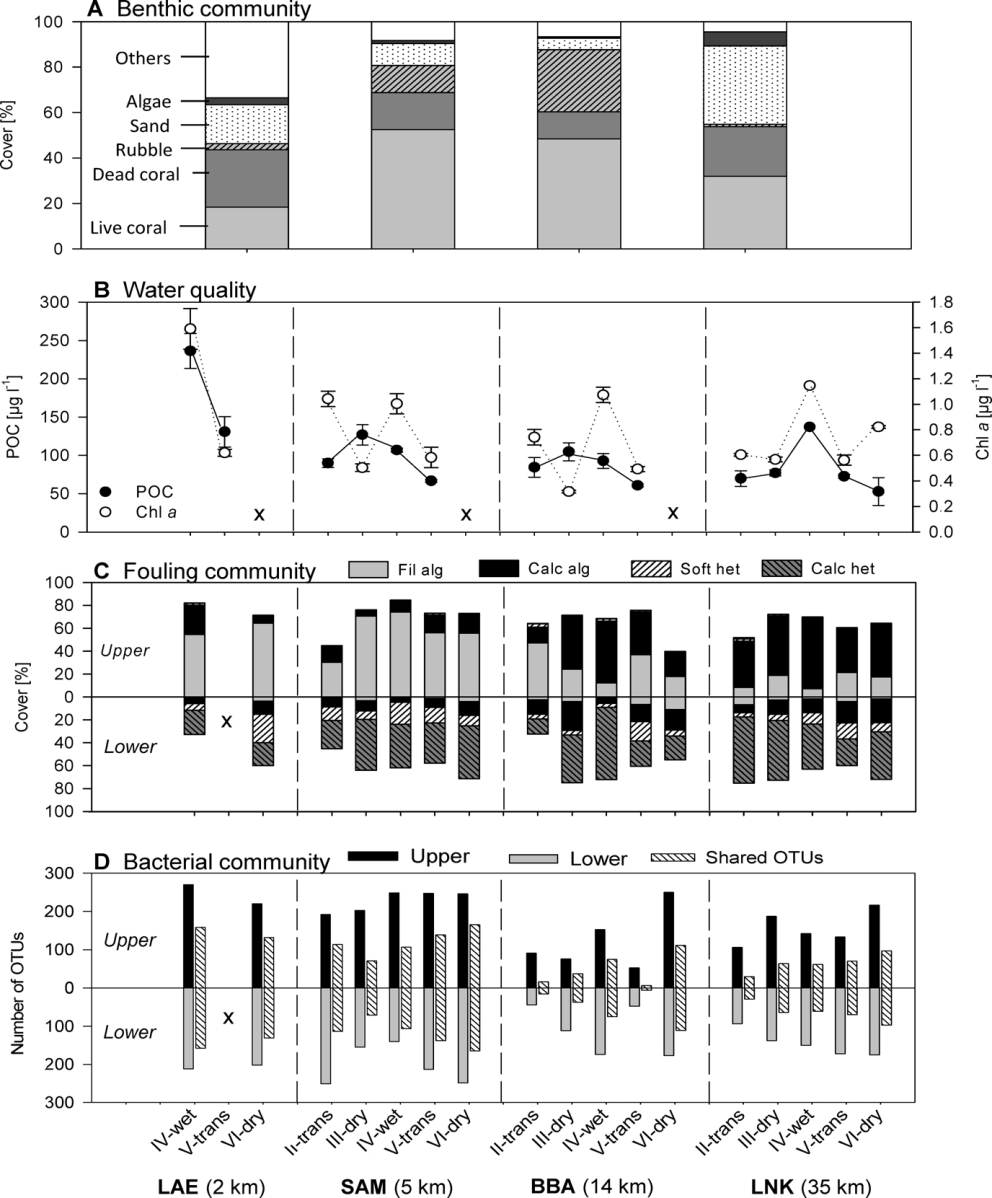
Spatio-temporal variations. (**A**) Benthic community structure at the four investigated sites. Spatial and seasonal patterns of (**B**) the major water parameter (mean ± SE): particulate organic carbon (POC) and chlorophyll *a* (Chl *a*), (**C**) the fouling community on the upper and lower face of the tile pairs: filamentous algae (Fil alg), crustose coralline red algae (CCA), soft heterotrophs (Soft het: sponges and ascidians) and calcareous heterotrophs (Calc het: barnacles, spirorbid worms and bivalves), (**D**) Bacterial OTU number on the upper and lower tile, and shared OTU numbers of the tile pairs. No data available (x).

The benthic community at the near-shore reef (site LAE) featured the lowest live coral cover (18.3%) and the highest proportion of dead coral (25.2%) and other organisms (33.5%) (Fig. 2A), especially soft corals (14.4%) and hydrozoans (9.1%) (Table S1). The highest live coral cover was found in the mid-shelf reefs (SAM: 52.5% and BBA: 48.4%), while off-shore LNK featured a live coral of 31.9% and a high cover by sand (34.5%) (Fig. 2A).

### Seasonal and Spatial Variations in Water Parameters

Seasonal fluctuations and spatial differences were reflected in the water parameters (Fig. 2B), with highest Chl *a* concentrations towards the end of the wet season (IV) at all sites, particularly in near-shore LAE waters (1.59±0.16 µg l^−1^) (mean ± SE) compared to mid-shelf and off-shore reefs (1.0±0.08 to 1.17 µg l^−1^) (in LNK season IV, only one water sample was available). Highest POC concentrations were also found in near-shore LAE at the end of the wet season (IV) (236±23 µg l^−1^) and peaked in the same season in off-shore LNK (137 µg l^−1^). The mid-shelf reefs SAM (127±13 µg l^−1^) and BBA (105±12 µg l^−1^) had a lower POC peak, which occurred towards the end of the dry season (III) (Fig. 2B). C/N ratios and DOC concentrations showed less pronounced seasonal and spatial patterns with C/N ratios between 5.7 and 9.8 and DOC concentrations between 52.2±7.1 and 147.1 µM (Table S2).

### Seasonal and Spatial Variations in the Macrofouling Community on tiles

**Figure 3:**
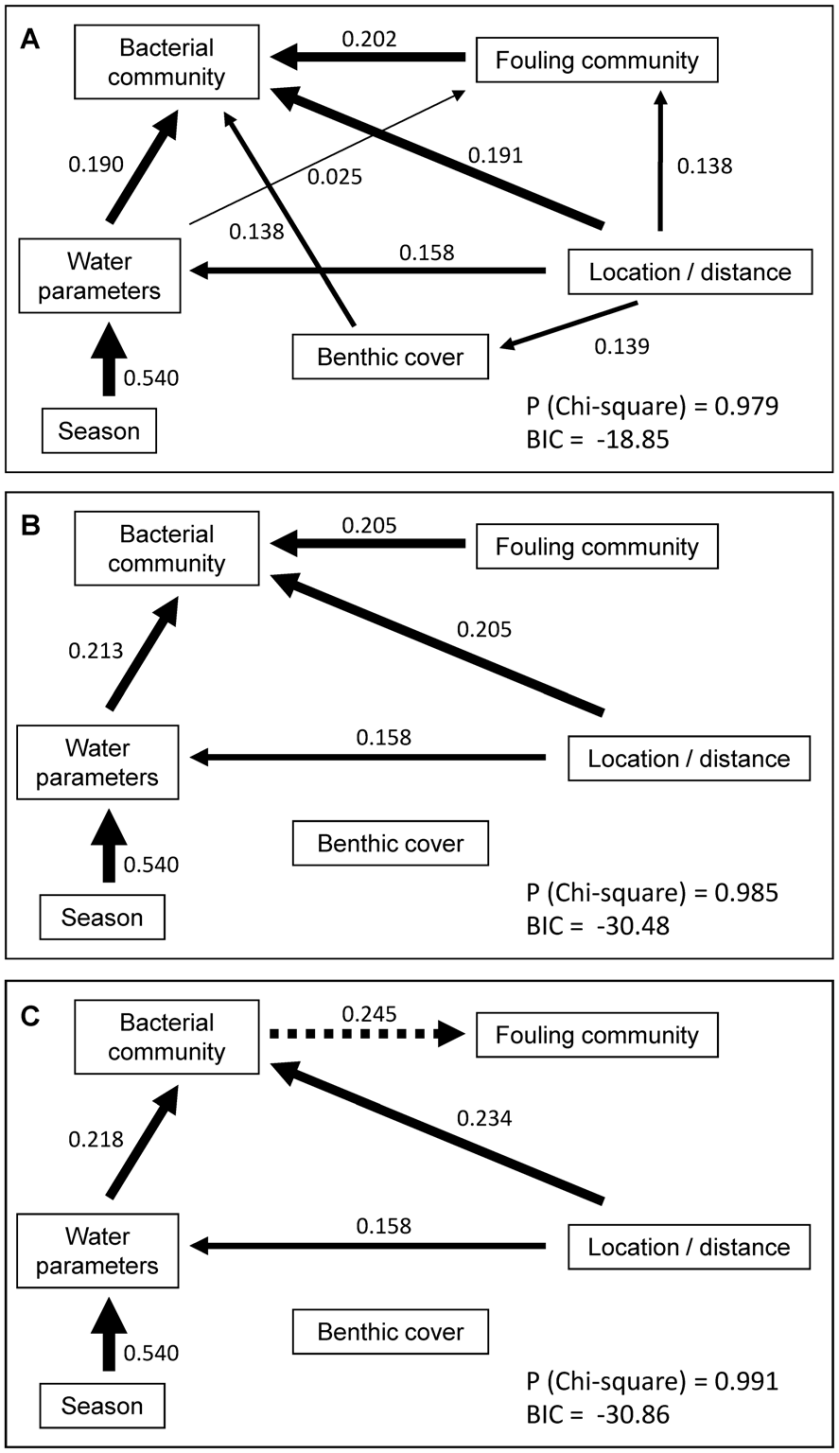
Results of path analysis including the initial (A), and final model 1 (B) and model 2 (C). In the final model 1 the path is directed from the fouling to the bacterial community (**B**) and in final model 2 from the bacterial to the fouling community (**C**). The significance value for the Chi-square test is given, assessing whether the model is significantly different from the corresponding correlation matrix. The Bayesian Information Criterion (BIC) is a measure of the goodness-of-fit and is minimized to obtain a better model fit. Numbers on each arrow indicate partial correlation coefficients associated with each causal relationship, and arrow thickness is also proportional to the partial correlation value.

For a total of 246 samples from tile pairs that were examined (not all tiles were available for recovery after the 4-months deployments), pronounced differences between the macrofouling community composition of the upper and lower sides were significant (ANOSIM R = 0.819, p = 0.001; Table S3) with a strong dominance of algae on the upper and of heterotrophs on lower sides, throughout the sites and seasons (Fig. 2C; Fig. S1). Spatial (site) effects were mainly found on the upper side (ANOSIM, R = 0.696, p = 0.001) with a visible shift from filamentous to crustose algae forms from the near- to off-shore sites (Fig. 2C). In contrast to the strong differences found between tile sides for the fouling community, no significant effects of water parameters, location or season were evidenced using multiple regression analyses (data not shown). These findings were corroborated by path analysis, where the best model found did not include a relationship between any of these factors and the fouling community (Fig. 3B and C).

### Seasonal and Spatial Variations in Bacterial OTU Numbers

On the 97 tile samples examined for bacterial diversity, a total of 445 OTUs were detected, with 400 OTUs occurring on more than 3 tiles. Overall, more OTUs were found on the upper side (356 OTUs on >3 tiles) compared to the lower side of the tiles (307 OTUs) (Fig. 2D), with 289 OTUs being shared between upper and lower sides. Overall, 352 OTUs occurred at all sites at least once within the 20-month period. Differences between the upper and lower side of the tiles were most pronounced in near-shore LAE and the overall OTU number was higher in the reefs close to shore (LAE and SAM) compared to mid-shelf BBA and off-shore LNK: The highest OTU number was found in near mid-shelf SAM (dry season VI: 330 OTUs), followed by near-shore LAE (wet season IV: 324 OTUs). The lowest OTU number was found in mid-shelf BBA (transition period V: 95 OTUs), which was also the site with highest seasonal variability, where OTU numbers varied between 95 (transition period V) and 316 (dry season VI) (Fig. 2D).

### Factors Influencing Bacterial Community Structure

**Figure 4:**
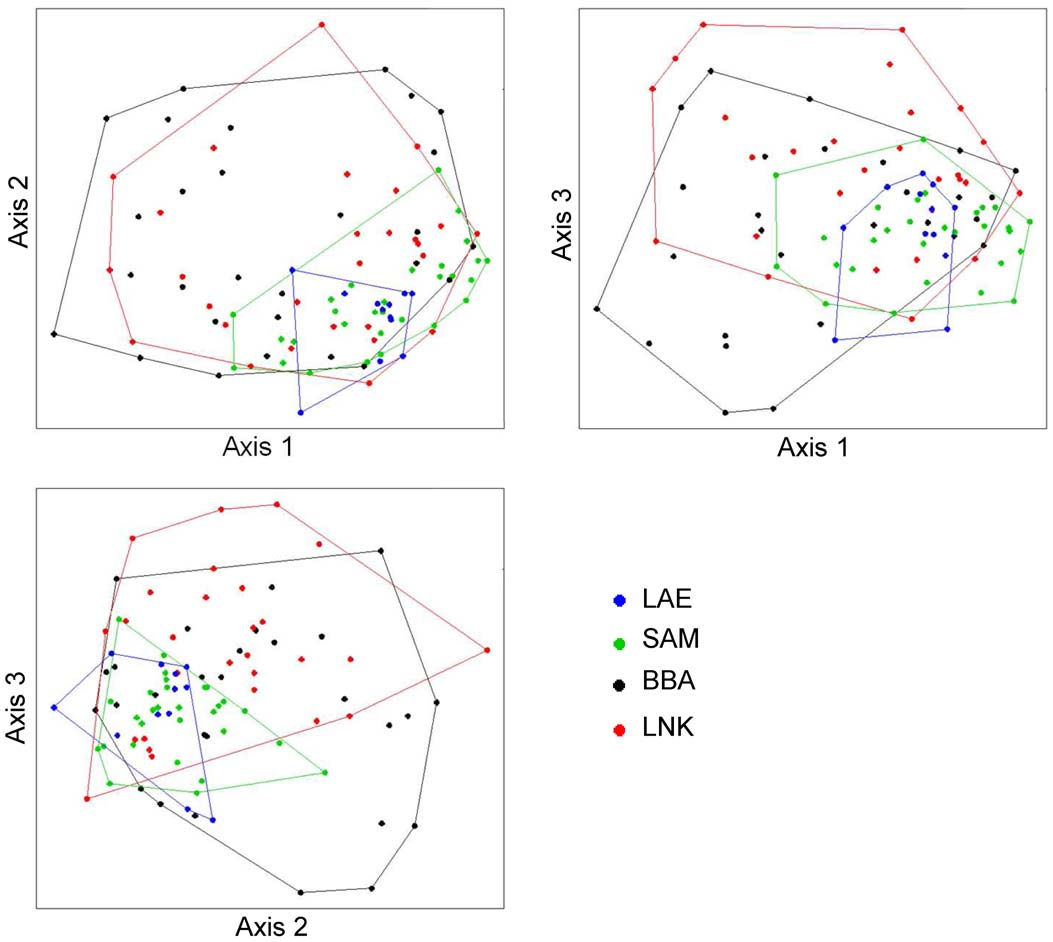
3D non-metric multidimensional scaling (MDS) plot of the Bray-Curtis-based dissimilarity matrix between bacterial communities colonizing the deployed tiles (dots) at the different sites. The colors used for polygons and the samples indicate the geographic positions with near-shore Lae Lae (LAE), near mid-shelf Samalona (SAM), far mid-shelf Bonebatang (BBA) and off-shore Lanyukan (LNK). Stress = 0.175.

Variation partitioning analysis was carried out to assess the individual and combined effects of contextual parameters on changes in bacterial community structure. Only data sets with complete contextual parameters were retained, thus excluding from the analyses, season VI in LAE, SAM and BBA, due to a lack of water parameters. Noticeably, each factor had a significant effect on the changes in bacterial community structure (Fig. S2), namely tile position (p<0.001 using 1000 permutations), season (p<0.001), site (p<0.001) and water parameter (p = 0.007), with generally between 1 and 3% of explained variance by the pure factor effects. Although the overall explained variance was rather low (8%, p<0.001), no covariation between factors was detected, indicating that no confounding effect was present among factors. Yet, when evaluating more specific comparisons using pairwise ANOSIM, the seasonal impact was found to greatly vary between sites, with the highest impact mid-shelf (BBA: R = 0.312, p<0.001) and lowest impact off-shore (LNK: R = 0.127, p<0.001). Furthermore, bacterial communities were most dissimilar during the wet season across all sites (IV: R = 0.363, p<0.001). The most striking finding was that bacterial community heterogeneity (as visualized by the spread of sample points within each site across seasons in the MDS plot; Fig. 4) was inversely related to the degree of site eutrophication, with the largest heterogeneity mid-shelf (BBA) and lowest near-shore (LAE; Fig. 4).

Because a large number of samples is generally required to carry out path analysis and, in our study the factor “tile position” was nested into the factor “site”, the finer distinction of upper and lower tile positions was not further considered. Path analysis was carried out to evaluate causal relationships between all factors (see Materials and Methods), with the initial model representing the most plausible relationships (Fig. 3A) as determined by our previous analyses (multiple regression analyses; see above). This resulted in a configuration (Bayesian Information Criterion [BIC], a measure of model fit and complexity, of −18.85), in which bacterial community structure was directly and mostly influenced by the macrofouling community (path coefficient of 0.20), environmental parameters (i.e. combined variation in particulate organic matter [POM], C/N ratio, dissolved organic carbon [DOC], and chlorophyll *a* [Chl *a*]; 0.19), location (0.19), and to a lesser extent by the benthic community (0.14). Environmental parameters were themselves mostly changing as a function of seasons (0.53) and, to a lesser degree, as a function of distance from shore (0.16).

After assessing the robustness of the initial configuration and removing less significant relationships, two best fitting models were found (BIC = −30.48 and −30.86, respectively): In Model 1 (Fig. 3B), water parameters were still largely influenced by seasonality (0.54). Bacterial community structure was equally influenced by the macrofouling community, the distance from shore and water parameters (all coefficients about 0.21). Model 2 (Fig. 3C) differed from Model 1 by the reversion of the arrow from bacterial community to macrofouling community (thus suggesting that changes in bacterial communities are the ones leading to subsequent changes in the fouling community), which resulted in a slightly better model fit with an increased site/distance effect (0.23) and a higher influence of the bacterial community on the macrofouling community (0.25) than in Model 1 (0.21).

**Figure 5:**
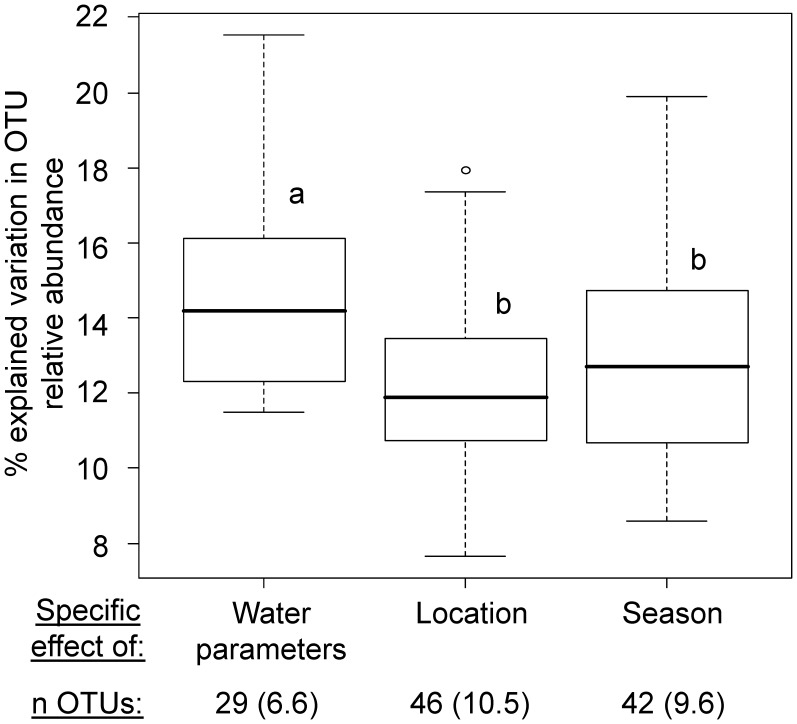
Detecting specialists vs. generalist OTUs by variation partitioning for individual OTUs. The numbers in italics indicate OTU numbers (percentage of the total of 437 OTUs), which variation in relative abundance was purely explained by the corresponding factor on the x-axis as determined by multivariate partial regression analyses, while taking all other factors into account. Different letters indicate significantly different means at p<0.05 (Welch two-sample *t*-test).

When performing variation partitioning at the individual OTU level to detect those OTUs that significantly responded to each contextual factor while taking the other factors into consideration, water parameters explained in general more of the variation (14%) than the other two factors (Fig. 5). Yet, those OTUs represented a relatively small fraction with 6.6% (29 OTUs) of the whole dataset as compared to the number of OTUs whose variations were purely explained by the two other factors (Fig. 5). Furthermore, environmentally, spatially or temporally controlled OTUs in the dataset were not necessarily the more abundant ones (as measured in relative fluorescence units) in general (Fig. S3). Although the effect of varying water parameters was most significant on certain OTUs, their small number (Fig. 5) associated with small to average relative abundances (Fig. S3) may explain the relatively small effect of water parameters on the overall community structure as determined by variation partitioning when the entire bacterial community was considered (see above).

## Discussion

The aim of this study was to assess bacterial diversity patterns of biofilms in coral reefs, their dynamics in relation to changes in the associated macrofouling community and their response to seasonal and spatial variations in environmental conditions. Changes in bacterial community structure associated with hard or sediment surfaces in higher latitude coral reefs have already been related to spatial differences in response to eutrophication [13], light availability (depth gradient) [19], wave energy [25] and seasonality [26], while the effect of associated macrofouling communities has not been addressed so far. Additionally, our study provides first data on the dynamics of bacterial communities in biofilms of equatorial coral reefs in the global centre of marine biodiversity [27]. Here, the investigated reefs were exposed to seasonal fluctuations mostly determined by the amount of nutrient supply through land run-off (rain fall) and to a lesser extent by temperature or light availability [28]. The results of our study revealed significant environmental effects, reflected in marked differences in bacterial community structure, which could be related to microhabitat, eutrophication, season, and to changes in the fouling community.

### Effect of Microhabitat (Sheltered vs. Exposed)

The microhabitats created by the tiles provided a sheltered/shaded and exposed/high light environment. They were found to be associated with the greatest differences in OTU number and significant changes in community structure of colonizing bacteria as compared to seasonal or regional influences. While the sheltered habitat was characterized by low light and a heterogeneous macrofouling community structure that would have provided a variety of surfaces for bacterial colonization, the exposed habitat was characterized by high light and a rather homogeneous algal cover. Interestingly, bacterial OTU number was higher on the exposed tiles, suggesting that bacterial communities may depend more on energy availability than on the offered substrate diversity. Organic material permanently settles on the upper tiles and is produced by the algae themselves, which may thus provide a large variety and quantity of nutrients for bacterial growth [29] and diversification [2], [15]. Additional reasons for a higher diversity on the exposed tiles may be a higher disturbance (e.g. grazing by fishes on the upper tiles), which continuously varies UV exposure (changes in UV may change bacterial communities; [30]) and thus creates space for new species to establish [6].

Although the high densities of cryptic filter-feeders are also known to harness significant amounts of organic material on both, artificial [31] and natural reef surfaces [32], thus potentially enriching this microhabitat, the reduced photosynthesis and lack of sedimentation may have reduced the overall supply of organic material to the microbial communities. In addition, the potentially antibacterial activity of the diverse secondary metabolites produced by macrofouling organisms (e.g. [20], [33]–[34]) may have contributed to the lower OTU numbers on the lower side of the tiles.

### Effect of Eutrophication

The eutrophication gradient was clearly evidenced in the macrofouling community structure by a shift from filamentous to crustose algae, which was previously described as an indicator for eutrophication [35]–[36]. Also the benthic community structure revealed strong signs of eutrophication and pollution in the most near-shore reef by a shift from hard corals to soft heterotrophic filter-feeding organisms [37]. However, the eutrophication gradient was less evidenced by the measured water parameters, although POC and Chl *a* have been found to be useful indicators in this area [38], most likely due to the localized and discontinuous nature of the water sampling campaign in this study.

Along the decreasing eutrophication gradient, bacterial communities were characterized by decreasing OTU numbers (Fig. 2D), but by increasing bacterial community heterogeneity (i.e. beta diversity; Fig. 4). Higher local bacterial diversity might be obtained in accordance to the energy-diversity relationships (e.g. [39]–[40]), which would favour high diversity under higher nutrient availability in near-shore reefs. This explanation would be further supported by our observations at the microhabitat level, where the bacterial community on the upper tiles experienced a stronger effect of eutrophication, since they harbour dense filamentous algae carpets that may serve as effective traps for sediment, particulate organic matter and associated pollutants in near-shore reefs.

The more similar community structure obtained in the eutrophied areas would be supported by previous observations of wider range of energy sources available in human impacted areas, which allow for more similar species to co-exist and for the selection of bacterial species that are generally more robust against the higher load of pollutants and toxins in eutrophied environments [15]. At the less impacted sites, the opposite processes would then be true, with less impact on local richness but more on the variation in community structure. From a technical viewpoint, it should also be noted that ARISA fingerprinting may not necessarily provide an accurate depiction of bacterial species richness, because e.g. the discrimination between bacterial types is based on ITS sequence length and ribosomal operon copy number may vary across microbial genomes [41], which would overall result in overestimating OTU number. Shifts in community structure based on ARISA community fingerprinting are yet useful to infer changes in diversity across samples and are generally in good agreement with patterns obtained by sequencing-based approaches of ribosomal genes (e.g. [26], [42]–[43]).

Changes in water quality can be crucial for some bacterial species and hence may limit their distribution. This seemed to be the case for 29 OTUs, which change in relative abundance and distribution was purely due to water parameters, regardless of variation in geographic location or seasons (Fig. 5). Such behaviour has been previously related to that of specialist species, while species purely affected by location and not by environmental conditions or seasonal fluctuations were considered generalist [44]. Here we followed this classification but, in addition, propose to also classify as generalists OTUs that were found at least once at each site regardless of season, tile side, or environmental conditions (n = 267 OTUs, from which 61 were already considered in the OTU numbers reported in Fig. 5). Due to limitations in detecting rarer OTUs, it should be mentioned that OTUs only found at a site may in fact have broader distribution range in the environment.

### Effect of Seasonality

Seasonality was clearly reflected in the variation of water parameters, bacterial community structure, but only weakly in changes of the fouling community. The OTU composition in mid-shelf reefs (SAM and BBA) revealed a pronounced seasonality, while the near- and off-shore reefs (LAE and LNK) featured a lower seasonality. This suggests a lower dependency of the bacterial community on substrate availability (i.e. fouling community), but rather a dependency on nutritional load. To explain those observations, we may thus propose the following scenario: At near-shore LAE, chronically high nutrient supply selects for specific bacterial community structure throughout the year. At near mid-shelf SAM, OTU numbers were high throughout the seasons and community structure was variable. This may be explained by a nutrient supply, which is comparatively high but still variable in its composition throughout the year, entailing variations in community structure. The oligotrophic mid-shelf BBA was associated with the largest differences in OTU number and community structure. Low OTU numbers were evidenced during the most nutrient-depleted period that occurs at the end of the transition period from wet to dry season, while tiles sampled in the dry season experienced occasional impacts of land run-off (rainfall) at the end of the season, which increased nutrient load levels and OTU number. At off-shore LNK, other oceanic factors (e.g. currents, upwelling) weakened the effects of monsoon related variability in nutrient supply (e.g. land run-off) that normally dominate on the shelf.

Although each factor was found to significantly affect changes in community structure when all parameters were included in the analyses, the overall low amount of explained community variation (variation partitioning) found in our study may be typical of classical community ecology studies (e.g. [45]–[46]). This is also consistent with the idea that environmental variables significantly explaining variation in community structure should be taken as putative ecological scenarios rather than as true reconstruction of the sources of variation under complex natural conditions.

### Relationships between Bacterial and Macrofouling Communities

Numerous studies have demonstrated the effects of biofilms on larval settlement by revealing large variations in species-specific responses and sensitivities (reviewed by [1]). A strong interplay between biofilm compositions and macrofouling has been suggested, with reciprocal effects between the biofilm community and settling macroorganisms [22]–[24]. Such reciprocal interactions between the two communities were also detected in our study by the path analysis approach, further justifying the use of complementary multivariate statistical approaches. However, the dependence was rather of weak magnitude due to differential responses of the two communities to external changes. Indeed, eutrophication clearly affected both macrofouling and bacterial communities, while seasonal fluctuations in environmental parameters only affected bacterial community structure, but hardly the macrofouling community structure.

Although we cannot exclude that the characterization of the two communities at different levels of taxonomic resolution might have masked some patterns of community covariation, the following reasons may most likely explain the low dependence: First, microorganisms may be expected to have much shorter generation times and larger population sizes than macroorganisms, which lead to much higher dynamics in bacterial than in macrofouling communities in response to environmental changes. Second, after 4 months of tile deployment, bacterial populations would have established their own microenvironments [2]–[3], which might have sheltered them from subsequent changes in the macrofouling community. The same may be valid for the macrofouling community, which also reaches an advanced state of succession after 4 months [47], allowing a certain independence from surface conditioning. Third, the bioactivity of secondary metabolites of the macrofouling community changes with seasons and this may affect microbial communities [48], while the macrofouling community structure may itself remain mostly unchanged.

In conclusion, this study identified bacterial patterns in biofilms of equatorial coral reefs and the likely factors that significantly affect them, be they variations in microhabitat, eutrophication level, seasonality or co-occurring macrofouling community. The complex interplay of all those factors was disentangled and resulted in new hypotheses: Not only nutrient availability and its seasonal fluctuations, but also the specific locations and degree of exposure, may be key parameters that shape bacterial community structure, diversity and ultimately functions. Because all factors had significant, yet modest contributions, other yet-unknown factors may also be at play in the study area and would need to be identified in the future (e.g. succession, secondary metabolites, other environmental parameters), as well as the impact of inter- and intra-species interactions within each of the investigated communities.

## Materials and Methods

### Study Sites and Sampling Design

Four reefs were sampled along a cross-shelf transect in the Spermonde Archipelago, Sulawesi (Indonesia) (Fig. 1), situated at the core of the Coral Triangle, the center of global marine biodiversity [49]. The archipelago consists of more than 100 small islands situated on a 40-km wide carbonate shelf surrounded by coral reefs. Due to environmental and ecological variability across the shelf, the archipelago has been divided into different ecological zones running parallel to the coast line [28], [50]. The near-shore zone, which includes the study site Lae Lae (LAE, 2 km distance from shore), is most strongly impacted by land run-off discharging waste water, fertilizers and sediments from the 1.5 million people harbor city Makassar and surroundings [28], [37]. This highly eutrophied, sediment loaded and polluted zone features the lowest diversity in various benthic reef taxa [51]. The mid-shelf zone includes the study sites Samalona (SAM, 5 km) and Bonebatang (BBA, 14 km). While SAM is still influenced by land run-off during the rainy season [28], BBA is situated in an oligotrophic environment beyond the reach of river plumes and features the highest diversity of corals [50] and other benthic reef taxa [51] in Spermonde Archipelago. The 4^th^ study site Lanyukan (LNK, 35 km) is situated on the off-shore edge of the Spermonde shelf bordering the deep Makassar Strait. Oceanic waters with moderate upwelling of nutrients [52]–[53] contribute to the lush development of coral reefs with intermediate levels of biodiversity [50].

The wet NW monsoon prevails from November to February (peak of rainfall: January, 730 mm month^−1^) and the dry SE monsoon from June to September (lowest rainfall: August, 15 mm month^−1^) (World Meteorological Organization). In this study, three seasons were considered, wet season (wet), transition from wet to dry season (trans) and dry season (dry), and consecutively labeled from II to VI: II (trans): March to June 2008; III (dry): July to October 2008; IV (wet): November 2008 to February 2009), V (trans): March to June 2009; VI (dry): July to October 2009.

Settlement tiles were deployed at 16 locations along the reef edge at about 3 m water depth, depth at which the highest live coral cover and reef diversity in the area are found [50]. A pair consisted of two terracotta tiles (15×15 cm) connected in the middle by a stainless steel bolt with unglazed sides facing outwards. The tile pairs were fixed on dead coral boulders in an angle of about 45° resulting in one upper (exposed) and one lower (sheltered) side. They were exchanged every 4 months between seasons. Sampling was performed underwater, while 3 pairs of tiles were chosen randomly and a small piece of each upper and lower tile was carefully broken and individually placed in small zip-lock bags. They were kept cool (max. 2 h) and subsequently frozen (−20°C) until microbial investigations were performed. The rest of the tiles and the remaining tiles were dried in the sun for the examination of the macrofouling community.

### Description of Macrofouling Community

Upper and lower sides of all tile pairs were inspected under a dissecting microscope. The following taxa of macrofouling community were distinguished: Filamentous algae, crustose coralline red algae, bryozoans, sponges, ascidians, barnacles, spirorbid worms and bivalves. The percentage cover of each taxon was recorded based on the visual estimation of total coverage of the upper or lower tile surface, respectively.

### Description of Benthic Community Composition of the Reefs

A 60-m line-intercept transect was conducted at each site along the reef edge in order to assess the benthic community composition of the reef [54]. A measuring tape was laid out along the transect and the underlying substrate was recorded on a cm-scale. The following categories were applied and calculated in percentage contribution: live coral (LC), dead coral (DC; >15 cm in diameter), coral rubble (RB; <15 cm), sand (SA), macroalgae (ALG) and others (OT), which included soft coral, sponge, anemone, ascidians and hydrozoans.

### Water Parameters

Water samples (n = 3) were taken with a 5-l Niskin bottle at the time of tile exchange at all sites. From each water sample, a 1-l subsample was filtered through a GF/F filter for chlorophyll *a* (Chl *a*) measurement and two 1-l subsamples were filtered through a pre-combusted and pre-weighed GF/F filter for analyses of total nitrogen and organic carbon of the particulate matter (particulate organic carbon: POC), respectively. Filters were stored at −20°C. A 10-ml sample of each filtrate was filled into a glass ampoule, acidified with H_3_PO_4_ (pH<2.0) and flame sealed for dissolved organic carbon (DOC) analyses. Chl *a* was extracted from the filter with 90% acetone over 24 h at 4°C, the sample was centrifuged (4000 rcf, 5 min) and measured fluorometrically (10-AU Fluorometer, Turner Design, CA) in a glass cuvette at an emission wavelength of 668 nm and an extinction wave length of 430 nm [55]. Calibration was carried out with a Chl *a* standard (Fluka, Sigma-Aldrich, Switzerland). Nitrogen and POC concentrations were measured with an elemental analyzer (NA2100 Protein, calibrated with CHNS standard [LECO]), while the filters for POC were acidified with 1 N HCl and dried prior analyses to remove the inorganic carbon. The C/N ratio was calculated by dividing the POC with the nitrogen value. DOC was measured via the combustion method with a total organic carbon analyzer (TOC-V_CPH_, Shimadzu) using low carbon and deep sea water standards (Hansell, RSMAS, Univ. of Miami).

### DNA Extraction and Automated rRNA Intergenic Spacer Analysis (ARISA)

An area of 2.25 cm^2^ (1.5×1.5 cm) on each tile was scraped with a scalpel. Microorganisms were suspended in 0.8 ml sodium phosphate buffer (120 mM, pH 8.0) and the suspension shaken (300 rpm, 10 min) and filtered through a 1.2 µm glass fiber filter (Type APFC, Millipore) to remove protists and eukaryotic cells [25], [56]. The filtrate was used for DNA extraction with the UltraClean soil DNA isolation kit (MoBio Laboratories, Inc., Carlsbad, CA, USA). Changes in the bacterial community structure were determined by ARISA [41] and ARISA-PCR were conducted in triplicates after the standard protocol described previously [57]. Briefly, 50-µl reactions were conducted with the universal primers ITSF (5′-GTCGTAACAAGGTAGCCGTA-3′) and eubacterial ITSReub (5′-GCCAAGGCATCCACC-3′), the latter being labeled with the phosphoramidite dye HEX (Biomers.net, Ulm, Germany). PCR cycling conditions consisted of an initial denaturation at 94°C for 3 min, followed by 30 cycles of 94°C for 45 s, 55°C for 45 s, 72°C for 30 s, and a final extension of 72°C for 5 min. PCR products were purified with Sephadex G-50 Superfine (Sigma-Aldrich, Germany) and the DNA concentrations were determined photometrically (Infinite 200 NanoQuant, Tecan). 100 ng of sample DNA were added to a separation cocktail containing 0.5 µl of internal size standard Map Marker 1000 Rox (50–1000 bp) (BioVentures, Inc., Washington D.C., USA) and 14.5 µl of deionized Hi-Di formamide (Applied Biosystems, Foster City, CA, USA) [57].

The amplified fragments were discriminated by capillary electrophoresis (Applied Biosystems, Foster City, CA). ARISA profiles were analyzed using the GeneMapper Software v3.7 (Applied Biosystems) and operational taxonomic units (OTUs) were identified for peaks with a minimum of 50 fluorescence units and OTU sizes between 100 and 1,000 bp. The GeneMapper output tables were further subjected to a “fixed window” binning strategy with a bin size of 2 bp [56]–[57]

### Statistical Analyses

General patterns in changes in bacterial and macrofouling communities considering sites, seasons and tile position were visualized by non-metric multidimensional scaling (MDS) ordination based on the Bray-Curtis dissimilarity matrix between samples. Analysis of Similarity (ANOSIM) was used to confirm the significance (p<0.05) of the community differences between groups of samples. The alpha criterion for significance was corrected for multiple pairwise comparisons using the Bonferroni correction criterion (see [58]–[59]). MDS and ANOSIM were performed with the multivariate statistic software PRIMER v6. Prior to performing further multivariate analyses, a consensus community profile was obtained for each sample by merging the triplicate PCR and by considering an OTU present if it appeared at least twice among the triplicates [57]. The merged table was Hellinger transformed to minimize the effects of the strongly left skewed distribution curve [60].

The specific effects of the main factors (distance from shore, season, tile position, water parameters, macrofouling community, benthic cover) and their co-variations on bacterial community structure were disentangled using “variation partitioning” and further explored in the deterministic modeling framework “path analysis” [61]. The effects of the overall model as well as those of each specific factor in variation partitioning were determined by simple and partial multivariate regression approaches and by testing their significance using 1000 Monte Carlo permutation tests. Those tests were carried out on with the statistical platform R (http://cran.r-project.org/) using the *vegan* package. Path analysis assesses the likelihood of different ecological scenarios (i.e. path models), which are represented by a network of dependencies between factors and/or variables, when confronted to the actual correlation structure of the data at hand [61]. Multivariate correlations between matrices were determined using the RV coefficient [62] applied to standardized data matrices, using the R package *FactoMineR*. The adequacy between the overall causal model and the original correlation matrix was assessed by Chi-square test, in which the p-value should not be significant, because the proposed model should be in good agreement with the data at hand and not reject it. The Bayesian Information Criterion (BIC, [63]) that measures model fit and complexity (*i.e.* a lower BIC value indicates a better fit of the model) was also used to evaluate model’s fit. The individual path coefficients (i.e. partial regression coefficients) that indicate the strength of the relation between causal and response variables and the fit of the overall path model were evaluated using the R package *sem*.

## Supporting Information

Figure S1 **Non-metric multidimensional scaling (MDS) of the fouling communities colonizing the deployed tiles at the different sites: near-shore Lae Lae (LAE), near mid-shelf Samalona (SAM), far mid-shelf Bonebatang (BBA) and off-shore Lanyukan (LNK).** The colors indicate the different seasons: transition period (trans: II & V), dry season (dry: III & VI) and wet season (wet: IV) on the upper (cross and star symbols) and lower tiles (filled symbols).
(TIF)

Figure S2 **Non-metric multidimensional scaling (MDS) of the bacterial communities colonizing the deployed tiles at the different sites: near-shore Lae Lae (LAE), near mid-shelf Samalona (SAM), far mid-shelf Bonebatang (BBA) and off-shore Lanyukan (LNK).** The colors indicate the different seasons: transition period (trans: II & V), dry season (dry: III & VI) and wet season (wet: IV) on the upper (cross and star symbols) and lower tiles (filled symbols).
(TIF)

Figure S3 **Abundance of OTUs, which are purely explained by the water parameters (red), the location (green) and by the seasons (blue).** The x axis represents the sum of relative fluorescence intensity for each OTU across all samples (i.e. its dominance in the dataset), while the y axis measures the frequency of each dominance class.
(TIF)

Table S1 Composition of the benthic community at the different sites.
(DOC)

Table S2 Water parameter at the different sites and during the different seasons.
(DOC)

Table S3 Results of Analysis of Similarity (ANOSIM) representing the spatial and seasonal patterns of the bacterial and fouling community.
(DOC)
