# Supplementary figures and images for: Effects of Eutrophication, Seasonality and Macrofouling on the Diversity of Bacterial Biofilms in Equatorial Coral Reefs
Source: PLoS One. 2012 Jul 6;7(7):e39951. doi: 10.1371/journal.pone.0039951 (PMC3391224; doi:10.1371/journal.pone.0039951)

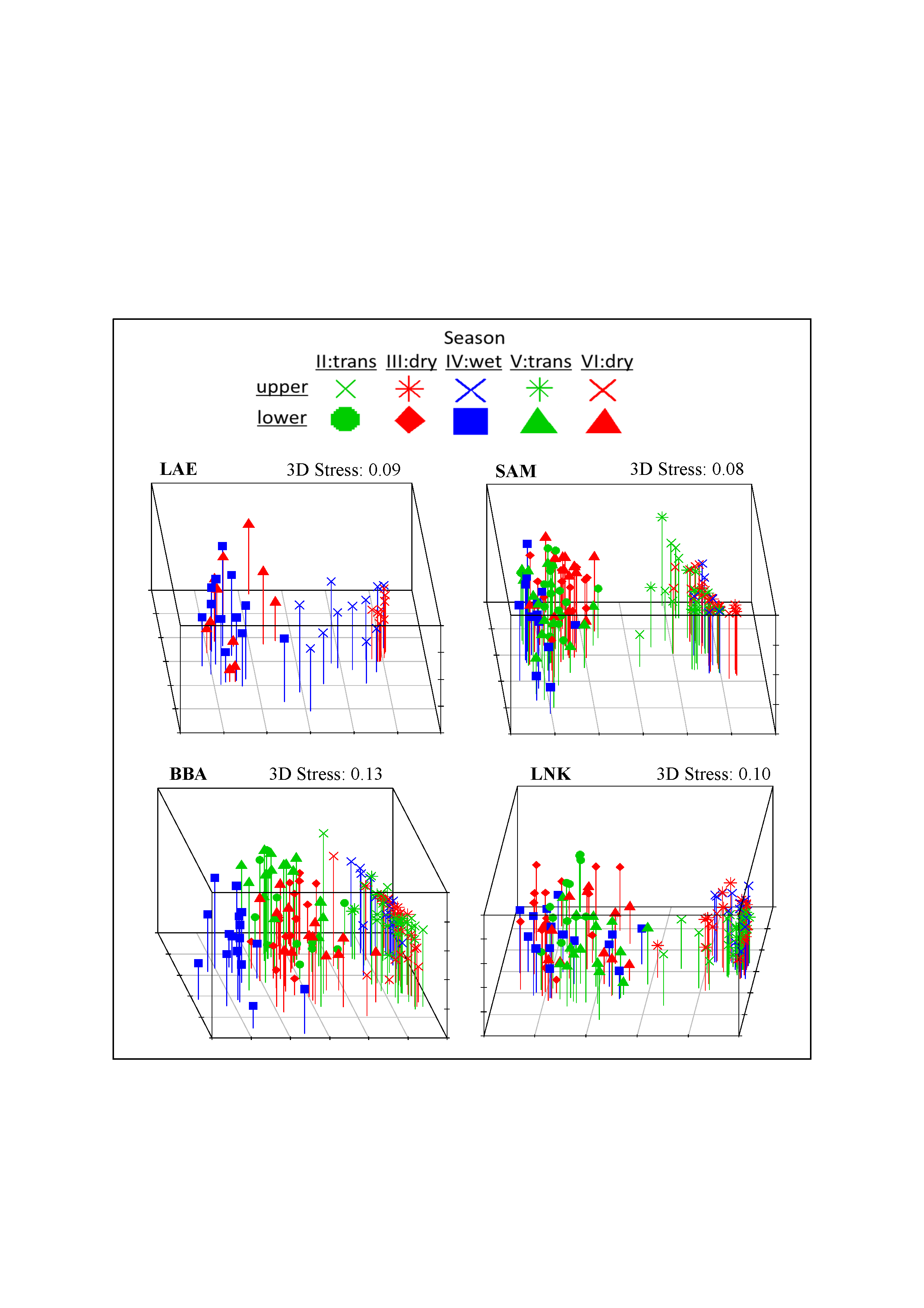

Supplement: Figure S1 — Non-metric multidimensional scaling (MDS) of the fouling communities colonizing the deployed tiles at the different sites: near-shore Lae Lae (LAE), near mid-shelf Samalona (SAM), far mid-shelf Bonebatang (BBA) and off-shore Lanyukan (LNK). The colors indicate the different seasons: transition period (trans: II & V), dry season (dry: III & VI) and wet season (wet: IV) on the upper (cross and star symbols) and lower tiles (filled symbols). (TIF) [file pone.0039951.s001.tif]

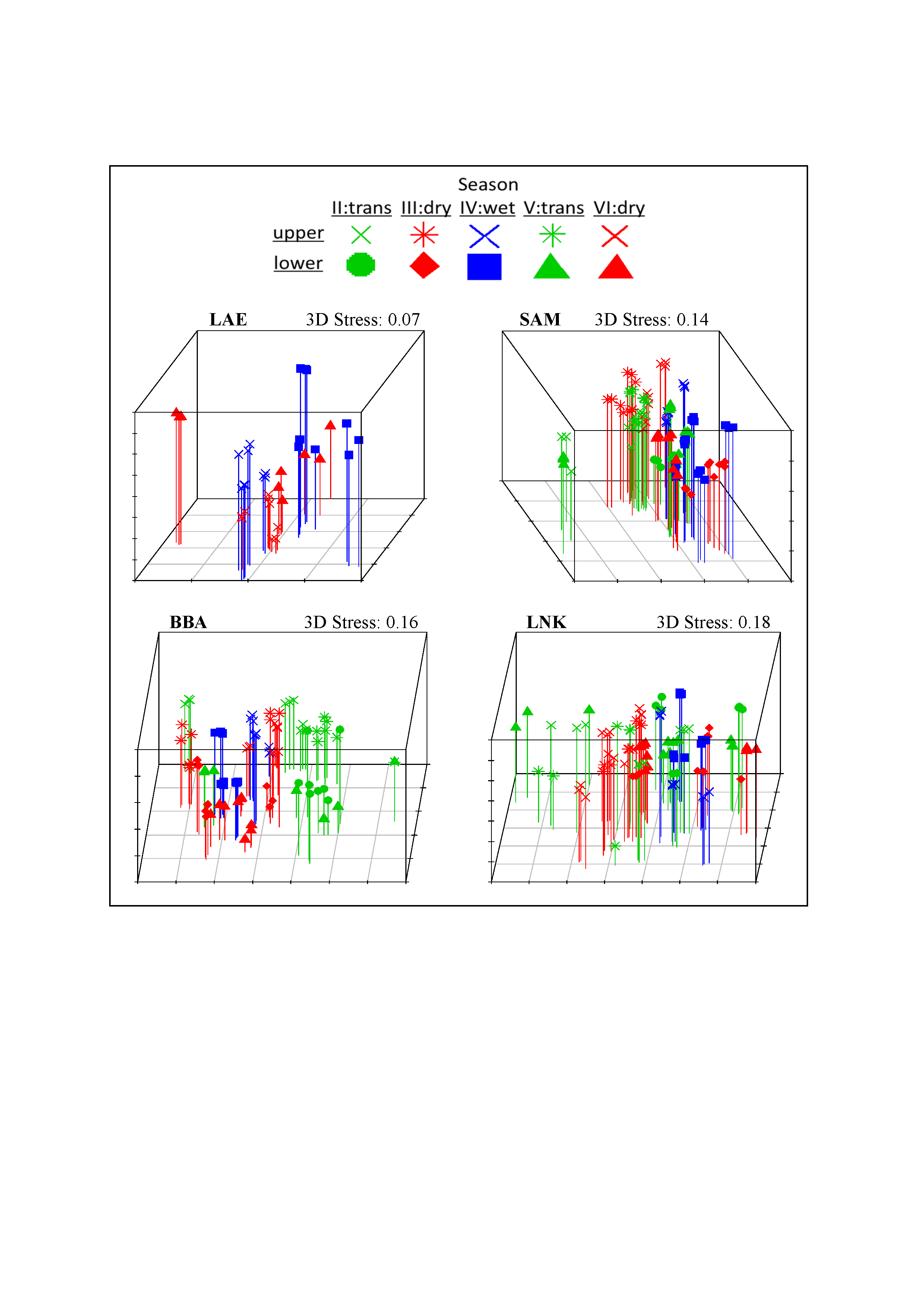

Supplement: Figure S2 — Non-metric multidimensional scaling (MDS) of the bacterial communities colonizing the deployed tiles at the different sites: near-shore Lae Lae (LAE), near mid-shelf Samalona (SAM), far mid-shelf Bonebatang (BBA) and off-shore Lanyukan (LNK). The colors indicate the different seasons: transition period (trans: II & V), dry season (dry: III & VI) and wet season (wet: IV) on the upper (cross and star symbols) and lower tiles (filled symbols). (TIF) [file pone.0039951.s002.tif]

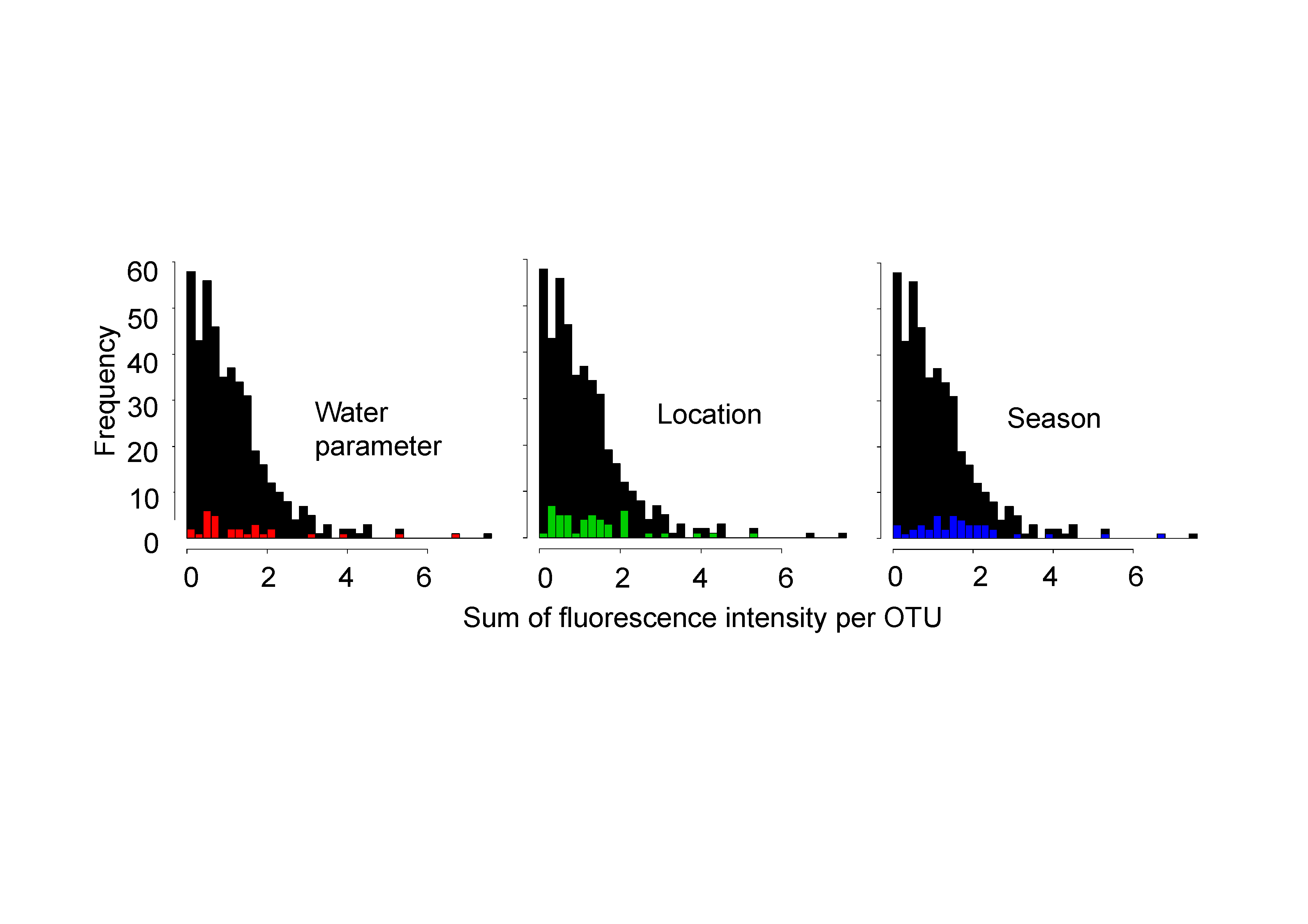

Supplement: Figure S3 — Abundance of OTUs, which are purely explained by the water parameters (red), the location (green) and by the seasons (blue). The x axis represents the sum of relative fluorescence intensity for each OTU across all samples (i.e. its dominance in the dataset), while the y axis measures the frequency of each dominance class. (TIF) [file pone.0039951.s003.tif]
