# Supplementary material for: Effects of Eutrophication, Seasonality and Macrofouling on the Diversity of Bacterial Biofilms in Equatorial Coral Reefs
Source: PLoS One. 2012 Jul 6;7(7):e39951. doi: 10.1371/journal.pone.0039951 (PMC3391224; doi:10.1371/journal.pone.0039951)
Supplement: Table S1 — Composition of the benthic community at the different sites. (DOC) [file pone.0039951.s004.doc]

Table S1: **Composition of the benthic community at the different sites.**
