# Supplementary material for: Effects of Eutrophication, Seasonality and Macrofouling on the Diversity of Bacterial Biofilms in Equatorial Coral Reefs
Source: PLoS One. 2012 Jul 6;7(7):e39951. doi: 10.1371/journal.pone.0039951 (PMC3391224; doi:10.1371/journal.pone.0039951)
Supplement: Table S2 — Water parameter at the different sites and during the different seasons. (DOC) [file pone.0039951.s005.doc]

Table S2: **Water parameter at the different sites and during the different seasons.**

Particulate organic carbon (POC), organic carbon / nitrogen ratio of particulate organic matter (C_org_/N), dissolved organic carbon (DOC) and chlorophyll a (chl *a*). Sites from near- to off-shore: Lae Lae (LAE), Samalona (SAM), Bonebatang (BBA) and Lanyukan (LNK). N=3, mean (SE), no data available (n. d.).
