# Supplementary material for: Effects of Eutrophication, Seasonality and Macrofouling on the Diversity of Bacterial Biofilms in Equatorial Coral Reefs
Source: PLoS One. 2012 Jul 6;7(7):e39951. doi: 10.1371/journal.pone.0039951 (PMC3391224; doi:10.1371/journal.pone.0039951)
Supplement: Table S3 — Results of Analysis of Similarity (ANOSIM) representing the spatial and seasonal patterns of the bacterial and fouling community. (DOC) [file pone.0039951.s006.doc]

Table S3: **Results of Analysis of Similarity (ANOSIM) representing the spatial and seasonal pattern of the bacterial and fouling community.**

(Global) R is a measure of community overlap with 0=completely overlapping and 1=completely dissimilar. Significant results are indicated by an asterisk: * p<0.05 and ** p<0.001 and R>0.2 in bold.
